# Stoichiometry and Turnover of the Bacterial Flagellar Switch Protein FliN

**DOI:** 10.1128/mBio.01216-14

**Published:** 2014-07-01

**Authors:** Nicolas J. Delalez, Richard M. Berry, Judith P. Armitage

**Affiliations:** ^a^ Biochemistry Department, University of Oxford, Oxford, United Kingdom; ^b^ Physics Department, University of Oxford, Oxford, United Kingdom; ^c^ Oxford Centre for Integrative Systems Biology, University of Oxford, Oxford, United Kingdom

## Abstract

Some proteins in biological complexes exchange with pools of free proteins while the complex is functioning. Evidence is emerging that protein exchange can be part of an adaptive mechanism. The bacterial flagellar motor is one of the most complex biological machines and is an ideal model system to study protein dynamics in large multimeric complexes. Recent studies showed that the copy number of FliM in the switch complex and the fraction of FliM that exchanges vary with the direction of flagellar rotation. Here, we investigated the stoichiometry and turnover of another switch complex component, FliN, labeled with the fluorescent protein CyPet, in *Escherichia coli*. Our results confirm that, *in vivo*, FliM and FliN form a complex with stoichiometry of 1:4 and function as a unit. We estimated that wild-type motors contained 120 ± 26 FliN molecules. Motors that rotated only clockwise (CW) or counterclockwise (CCW) contained 114 ± 17 and 144 ± 26 FliN molecules, respectively. The ratio of CCW-to-CW FliN copy numbers was 1.26, very close to that of 1.29 reported previously for FliM. We also measured the exchange of FliN molecules, which had a time scale and dependence upon rotation direction similar to those of FliM, consistent with an exchange of FliM-FliN as a unit. Our work confirms the highly dynamic nature of multimeric protein complexes and indicates that, under physiological conditions, these machines might not be the stable, complete structures suggested by averaged fixed methodologies but, rather, incomplete rings that can respond and adapt to changing environments.

## IMPORTANCE

The flagellum is one of the most complex structures in a bacterial cell, with the core motor proteins conserved across species. Evidence is now emerging that turnover of some of these motor proteins depends on motor activity, suggesting that turnover is important for function. The switch complex transmits the chemosensory signal to the rotor, and we show, by using single-cell measurement, that both the copy number and the fraction of exchanging molecules vary with the rotational bias of the rotor. When the motor is locked in counterclockwise rotation, the copy number is similar to that determined by averaged, fixed methodologies, but when locked in a clockwise direction, the number is much lower, suggesting that that the switch complex ring is incomplete. Our results suggest that motor remodeling is an important component in tuning responses and adaptation at the motor.

## INTRODUCTION

The bacterial flagellar motor is one of the most complex biological machines and a model system for the investigation of protein dynamics and turnover in functioning multimeric protein complexes (1). Its rotation direction is regulated by a sensory pathway, allowing bacteria to navigate their environment in search of better conditions (2). The *Escherichia coli* flagellar motor is ~50 nm in diameter, embedded in the cell membrane, and drives a long, helical filament that is several micrometers long. The basal body, which is the rotor at the core of the motor, consists of a set of rings and is surrounded by a ring of stator units that generate torque using transmembrane ion flux (3, 4). Recent studies have shown that functioning motors undergo adaptive remodeling in response to intra- and extracellular signals, casting a new light on the role of protein exchange in the functioning of macromolecular complexes (5–11).

The C ring, also called the switch complex, forms the cytoplasmic part of the rotor. It is composed of three proteins: FliG, FliM, and FliN (1). Binding of the chemotaxis response regulator CheY in its phosphorylated form (CheY-P) to the N-terminal segment of FliM and, subsequently, to FliN (12), increases the probability that the *E. coli* motor will switch from counterclockwise (CCW) to clockwise (CW) rotation (13–16). Recently, it was shown that FliM molecules undergo rapid stochastic exchange between the rotor and the cytoplasm while the motor is functioning. The fraction of FliM that exchanges varies with the rotational bias, increasing from 0.26 in CCW motors to 0.52 in CW motors (6, 8). Stepwise photobleaching experiments showed that the number of FliM molecules in wild-type motors is 30 ± 6. This number was also found to vary with the rotational bias of the motor: in Δ*cheY* cells, in which the motors are CCW-locked, the mean number of FliM molecules per motor was found to be 1.29 times larger than in cells expressing CW-locked motors. This does not agree with previous three-dimensional cryoelectron microscopy reconstructions of rotors, which revealed a 32- to 36-fold symmetry of the C ring regardless of the rotational bias of the motor (17–19). Most motors were found to have a 34-fold symmetry, and the 32- to 36-fold range corresponded either to variations in rotor diameter or, occasionally, to gaps in the ring. However, the ratio of 1.29 between the average numbers of FliM molecules seen by fluorescence microscopy in CCW versus CW motors is larger than the range observed by electron microscopy (EM) to date (1.12), raising the question of how a CCW motor accommodates the extra molecules in the C ring.

**FIG 1:**
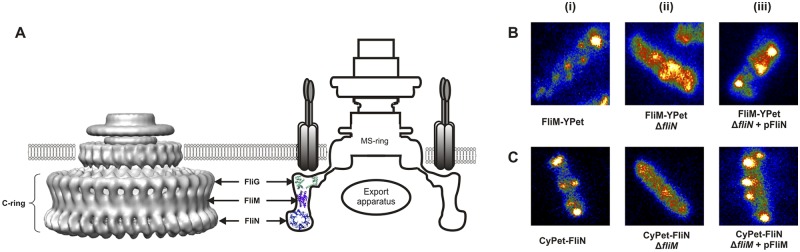
(A) Three-dimensional reconstructions from cryoelectron micrographs of the CW-locked rotor from *Salmonella enterica* serovar Typhimurium (PDB EMDB accession number 1887) (left) (19), and schematic of the motor structure (right). Three different proteins compose the C ring: FliG, FliM, and FliN. (B) (i) FliM-YPet complexes in wild-type cells. (ii) Some complexes can still be seen in Δ*fliN* cells, but the cells have a higher fluorescent background. (iii) Expression of *fliN* from an inducible plasmid restores wild-type pattern. (C) (i) CyPet-FliN complexes in wild-type cells. (ii) No spots can be seen when *fliM* is deleted. (iii) Expression of *fliM* restores wild-type pattern.

With more than 100 molecules per rotor, FliN is a major component of the switch complex, although its precise role remains unclear. *In vitro* biochemical and structural studies have shown that FliN molecules are organized in doughnut-shaped tetramers that bind to the C-terminal region of FliM, forming an array of alternating FliM and FliN molecules at the bottom of the C ring (Fig. 1A) (20–22). FliN is also thought to be involved in flagellum-specific export by providing binding sites for the chaperone-substrate complexes for their concentration near the export gate. Here, we investigated the stoichiometry and turnover in *E. coli* of FliN labeled with CyPet (CFP homolog) and compared our findings with data on FliM.

## RESULTS

All analysis of CyPet-MotB and of CyPet-FliN motors (except for FliM/N complementation experiments) were performed using epifluorescence microscopy of fluorescent spots that were at the center of rotation of a tethered *E. coli* cell (see Video S1 in the supplemental material), ensuring that we analyzed only functioning motors.

### FliN stabilizes FliM in the C ring.

FliN molecules sit at the bottom of the C ring, where they self-assemble in tetramers which in turn bind to FliM. Li and Sourjik (23) reported that FliM can still bind the basal body in the absence of FliN but FliN requires FliM to bind to the basal body. We confirmed these observations by looking at FliM-YPet/Δ*fliN* and CyPet-FliN/Δ*fliM E. coli* cells (Fig. 1B and C). Consistent with the earlier report (23), we found that FliM-YPet (YFP homolog) complexes were still observed in the absence of FliN proteins, whereas no CyPet-FliN spots could be observed when *fliM* was deleted. Upon expression of the deleted gene from an inducible plasmid, fluorescent spots showed a wild-type pattern. However, the higher cytoplasmic fluorescence background and reduced intensity of motor spots observed in the FliM-YPet/Δ*fliN* strain suggests that the anchoring of FliM to FliG is stabilized by the presence of FliN. These results indicate that FliN tetramers anchor to and stabilize the FliM ring.

### FliN copy numbers in wild-type motors.

**FIG 2:**
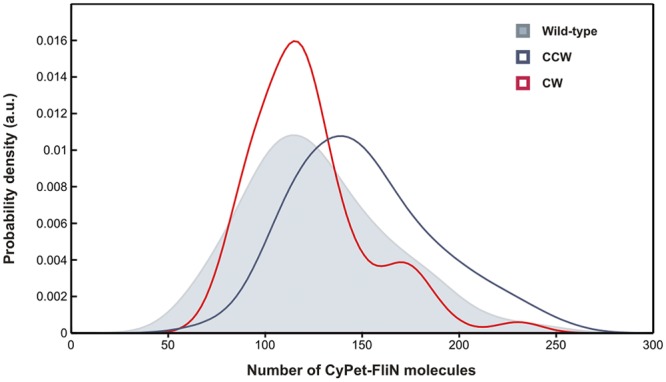
CyPet-FliN distribution in wild-type motors (shaded curve, 80 cells), CW motors (red solid line, 55 cells), and CCW motors (blue solid line, 64 cells). Gaussian kernel distribution estimations peaked at 120 ± 26 for wild type, 114 ± 17 for CW and 144 ± 26 for CCW motors.

Direct quantitative measurement of protein stoichiometry by stepwise photobleaching is not a suitable method for components with high copy numbers, such as FliN. Therefore, we used a motor component with known stoichiometry, MotB, as a reference, comparing the fluorescence intensities from individual CyPet-FliN motors to those of CyPet-MotB motors imaged using epifluorescence microscopy under identical illumination and imaging conditions (see Materials and Methods). MotB is a stator protein that contains a C-terminal periplasmic domain that binds the cell wall and an N-terminal membrane-spanning domain that extends into the cytoplasm. Its average copy number in tethered cells, i.e., at high load, was shown to be 22 (5). We therefore tagged MotB with CyPet as described previously (5), ensuring that the fluorescent tag was located in the cytoplasm to allow direct comparison with CyPet-FliN. Figure 2 shows the distribution of the number of FliN molecules estimated for each motor spot in *E. coli* cells in which genomic *fliN* was replaced with *cyPet-fliN*. The Gaussian fit to the unbiased kernel density estimation of the distribution peaked at 120 ± 26 CyPet-FliN molecules for wild-type motors. This number suggests a 1:4 FliM:FliN stoichiometry, as 30 ± 6 FliM copies were measured in wild-type motors in a previous experiment (6), confirming previous *in vitro* estimates (21). The long tail of bright spots in the distribution may be due in part to pairs of motors that are too close together to be resolved (Fig. 2).

### FliN copy number varies with the rotational bias of the motor.

Lele et al. recently observed that the number of FliM molecules associated with the motor was on average 1.29 times larger in the CCW state than in the CW state (8). We therefore investigated whether FliN molecules follow the same pattern. We measured CyPet-FliN copy numbers in Δ*cheY* cells (CCW-locked) and Δ*cheY* cells complemented with the constitutively active CheY mutant CheY^D13K/Y106W^ (CW-locked), using the same method as with the wild-type strain. The Gaussian fits on the distributions peaked at 144 ± 26 counts for CCW motors and 114 ± 17 counts for CW motors (Fig. 2). This gives a ratio of 1.26 between the mean numbers of FliN molecules present in the C ring in CCW versus CW motors, in good agreement with the value found for FliM. These results suggest further that the 1:4 FliM:FliN complex works as a unit within the C ring, implying that the average number of FliM molecules in the motor is 28 ± 4 in a CW motor and 36 ± 6 in a CCW motor. We also complemented the *cyPet-fliN*/Δ*cheY* strain with the CheY mutant CheY^D57A^ that cannot be phosphorylated and performed the same experiment. In this case, the Gaussian fits on the distributions peaked at 143 ± 30 CyPet-FliN molecules, similar to the value found for CCW motors (see Fig. S2 in the supplemental material).

### FliN exchange.

**FIG 3:**
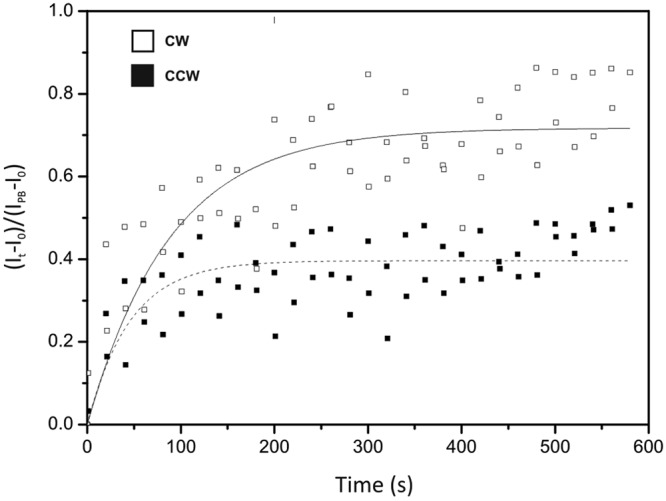
Average fluorescence recovery versus time for CyPet-FliN in CCW (*n* = 8) and CW (*n* = 17) motors. Values for *k*_off_ were obtained from the following fits: 0.022 ± 0.005 s^−1^ for CCW and 0.011 ± 0.002 s^−1^ for CW. Recovery fractions of CyPet-FliN molecules were 0.39 ± 0.012 for CCW and 0.71 ± 0.022 for CW motors.

The results described above suggest that FliM and FliN form a complex with stoichiometry of 1:4 when bound to the motor. FliM has been shown to exist in two discrete populations within the C ring, one tightly associated with the motor and the other undergoing stochastic exchange (6). The ratio between these two FliM populations varies with the rotational bias of the motor, with the fraction of exchanging molecules increasing when the motor rotates in a clockwise direction (6, 8). We therefore investigated whether FliN dynamics are similar to FliM dynamics by performing fluorescence recovery after photobleaching (FRAP) experiments on rotating CyPet-FliN motors with different CW biases. We bleached the fluorescence in the motors with a focused laser spot and measured recovery by taking two 140-ms snapshots every 20 s for 580 s. Measurements of the increase in intensity following photobleaching (*I*_*t*_ − *I*_0_, where *I*_*t*_ is the intensity of the motor spot at time *t* after bleaching and *I*_0_ the intensity directly after bleaching) were normalized by *I*_PB_ − *I*_0_, with *I*_PB_ being the intensity before bleaching averaged over 10 data points. Every value was also corrected by a photobleaching factor, exp(*t*_tot_/τ), as determined previously (5), with *t*_tot_ being the total time spent under illumination and τ the time constant, measured under our experimental conditions at τ = 44.68 s (*n* = 20 traces). The average FRAP traces for the two strains are shown in Fig. 3. Exponential fits to the recovery traces (8) showed 39% ± 1.2% and 71% ± 2.2% fluorescence recovery for CCW and CW motors, respectively. The recovery time constants were 45 ± 10 s for CCW motors and 90 ± 16 s for CW motors. Note that the absolute initial rates of recovery are approximately the same for CW and CCW motors. Approximately 10% of all the FliN molecules in the cell were bleached by the laser spot (see Materials and Methods), indicating that not all the proteins exchange and that two FliN populations exist in the motor. These numbers are comparable to those measured for FliM, within experimental error, suggesting further that FliM and FliN are tightly associated with each other in the C ring and that the FliM_1_:FliN_4_ protomer works as a unit.

## DISCUSSION

Our estimate of ~120 FliN molecules in wild-type motors is in very good agreement with our previous measurements of FliM copy numbers, assuming a 4:1 ratio (Fig. 2). This result confirms previous *in vitro* biochemical studies reporting the self-assembly of FliN molecules as tetramers (21), which in turn bind to FliM to form a FliM_1_:FliN_4_ protomer. Our data also confirm that FliN molecules do not self-assemble in large complexes (Fig. 1C) but, rather, bind to and stabilize FliM within the C ring (Fig. 1B) (23). Stabilization of FliM by FliN (Fig. 1B) is consistent with the nonmotile phenotype of Δ*fliN* cells.

The mean numbers of CyPet-FliN molecules were 114 ± 17 in CW cells and 144 ± 26 in CCW cells, a ratio of 1.26, very close to that of 1.29 reported previously for FliM (8). In addition, our FRAP experiments showed that the fraction of exchanging FliN molecules varies with the rotational bias of the motor, similarly to FliM (6, 8), the exchanging population was larger in CW motors than in CCW motors, suggesting an exchange of FliM_1_:FliN_4_ as a unit. Given that ~10% of all the CyPet-FliN molecules in the cell were bleached by the focused laser pulse, 71% (39%) fluorescence recovery corresponds to 79% (43%) exchange of CyPet-FliN molecules in a CW (CCW) motor. The rates of exchange for CW and CCW motors also matched those found previously for FliM, within experimental error (6, 8). Our results, therefore, suggest that the FliM_1_:FliN_4_ complex works as a unit within the C ring. We occasionally observed moving CyPet-FliN spots (see Videos S2 and S3 in the supplemental material) with intensities corresponding to 20 to 80 molecules which were not seen in the Δ*fliM* strain. The nature of these spots is unknown. Possibilities include incomplete switch complexes not yet anchored to the cell envelope or other, unknown multimeric complexes containing FliM and FliN, suggesting that the FliM:FliN protomers exist in the cytoplasm when not bound to the rotor.

Three-dimensional reconstructions of the rotor from electron cryomicrographs have consistently reported a 32- to 36-fold symmetry for the C ring, regardless of the rotational bias. Lele et al. proposed that the symmetry observed by EM (~34) corresponds to the minimum number of FliM bound to the rotor (i.e., in CW motors), meaning that CCW motors would have an average of ~44 FliM copies (8). This raises the questions of why such a stoichiometry has never been observed by EM and how the C ring would accommodate the extra molecules. It is possible that subunit dissociation, but not replacement, continues during sample preparation for EM. Ring shrinkage would then occur until a relatively stable situation is reached. However, what would confer stability to a ring with 34-fold symmetry rather than a ring with a different symmetry, e.g., the 26-fold symmetry of the MS ring, is unclear. Our quantification of the absolute numbers of FliN molecules in CW and CCW motors is based on the assumptions that the fluorescence intensity of a CyPet molecule is the same whether fused to FliN or MotB and that the average number of fluorescently labeled MotB molecules per motor is 22, as determined previously (5). The recent observation that the number of stator units anchored in a motor depends upon external load (10, 11) is not expected to affect the latter assumption, as we measured CyPet-MotB spot intensities in tethered cells under the same high load as for the previous estimate of 22 per motor, where the number of MotB molecules is expected to be maximal. If our estimates are correct and if there are 4 FliN copies and one FliM copy per repeating unit of the C ring, as shown by the large amount of biochemical evidence (20, 21), then the number of units in the ring ranges from 28 ± 4 in CW motors to 36 ± 7 in CCW motors. A smaller number of MotB molecules per motor would shift this range likewise, e.g., an assumption of 20 MotB molecules would give a range of 26 ± 4 units in CW motors and 33 ± 6 units in CCW motors. One possible explanation of how this variability can be reconciled with the observed 32- to 36-fold symmetry observed by electron microscopy is that CCW motors have complete rings, with one FliM_1_:FliN_4_ unit bound to each of ~34 FliG molecules, and wild-type or CW motors contain gaps where entire FliM_1_:FliN_4_ units are missing. Gaps in the C ring were occasionally observed by EM but were thought at the time to result from the purification conditions (16). A careful analysis of single EM images might reveal that incomplete structures are more common than expected. CW-locked motors are ideal candidates to test this idea. Our model of an incomplete FliM:FliN ring is incompatible with the conformational spread model as a mechanism for cooperativity in the flagellar switch which has recently been observed experimentally. This suggests that the FliG ring is the most likely candidate for cooperativity and should therefore not contain any gaps. Information on the stoichiometry and turnover of FliG is required for a better understanding of the mechanism of the flagellar motor.

## MATERIALS AND METHODS

### Cell strains and preparation.

Four experimental strains were used for this study (Table 1). Cells were grown in 10 ml tryptone broth (TB) at 30°C to mid-log phase (optical density at 600 nm of 0.5). When needed, 100 µM of isopropyl-β-d-thiogalactopyranoside (IPTG) and 100 µl·ml^−1^ of ampicillin were added to the TB. Filaments were sheared by forcing 1 ml of the cell suspension 75 times between two syringes with 26-gauge needles connected by a piece of polyethylene tubing (12 cm long, 0.58-mm inner diameter) (12). The cell suspension was centrifuged 3 times at 6,000 × *g* for 3 min and resuspended in 75 µl motility buffer. The cells were flowed through a tunnel slide and left to incubate for 5 min. Motility buffer containing 50 µl·ml^−1^ of chloramphenicol was then flushed through the tunnel slide to remove unbound cells.

**TABLE 1:** List of strains used in study

Strain	Description^*a*^	Background
JPA809	*cyPet-fliN fliC*(St)	RP437 (24)^*b*^
JPA810	*cyPet-fliN fliC*(St) Δ*cheY*	RP5232 (24)
JPA811	*cyPet-fliN fliC*(St) Δ*cheY*(pIND-cheY^D13K/Y106W^)	RP5232
JPA812	*cyPet-fliN fliC*(St) Δ*cheY*(pIND-cheY^D57A^)	RP437
JPA813	*cyPet-motB fliC*(St)	RP437

^a^ St, sticky phenotype.

^b^ Reference for background strains.

### Microscopy.

We used a home-built inverted microscope with a 15-mW 440-nm laser (PPMT LD1650; Laser 2000, United Kingdom) and a 532-nm diode-pumped solid-state (DPSS) laser (Laser 2000), as described previously (6). Laser epifluorescence illumination was used for all fluorescence imaging of the motor spots for measurements of the copy number of CyPet-FliN molecules, and total internal reflection fluorescence (TIRF) mode for complementation experiments. The intensities were ~0.127 µW·µm^−2^ and ~2 µW·µm^−2^ for the 440-nm (CyPet-MotB and CyPet-FliN) and 532-nm (FliM-YPet) illumination, respectively. Photobleaching of the CyPet-FliN motors was achieved by a 420-ms exposure to a focused laser spot (~3 mW·µm^−2^) centered over the fluorescent spot at the center of rotation of the tethered cell. The total intensity integrated over the whole cell immediately after this photobleaching was 90% ± 4% of that before photobleaching.

Fluorescence emissions from CyPet-FliN and YPet-FliM were imaged, as described previously (6), in frame transfer mode at 50 nm/pixel at 25 Hz using a 128- by 128-pixel, cooled, back-thinned electron-multiplying charge-coupled device camera (iXon DV860-BI; Andor Technology). Fifty frames were recorded for stoichiometry experiments, and the initial fluorescence intensity was extracted using the method described previously (6, 10). For complementation experiments examining the effect of FliM and FliN on each other, a single frame was recorded for each motor. For FRAP experiments, two 140-ms epifluorescence exposures were taken every 20 s after photobleaching.

CyPet-MotB motors were ~7 times less bright than CyPet-FliN motors and showed the expected linear relationship between exposure time and fluorescence intensity (see Fig. S1 in the supplemental material). Therefore, the exposure time was increased 7-fold for observations of CyPet-MotB spots.

### Image acquisition and analysis.

The fluorescence intensity of each motor spot was determined manually using ImageJ for each frame. Average curves were generated for FRAP, and all intensity components were corrected for photobleaching during observation by multiplication with a cumulative factor, exp(*t*_total_/*t*_0_), where *t*_total_ is the total accumulated time under observation and *t*_0_ is the appropriate bleach time constant measured over 35 cells under the conditions described above (*t*_0_ = 44.68 s). The prebleach fluorescence intensity (*I*_PB_) was determined by averaging over 10 frames before bleaching. Fluorescence recovery data were fitted using the BoxLucas1 fitting option in OriginPro 8.5.1 (OriginLab Corporation, USA).

### Estimating FliN stoichiometry.

The fluorescence intensity of a single CyPet molecule was estimated using CyPet-MotB as follows. The initial intensities of 45 CyPet-MotB motor spots were measured, and the average spot intensity at a 40-ms exposure time (i.e., under imaging conditions identical to those used for CyPet-FliN) was found to be 745 ± 112 counts (see Fig. S1 in the supplemental material). Assuming an average of 22 MotB molecules per motor (5), we estimated that each CyPet-MotB molecule contributes 33 ± 8 counts to the intensity of a motor spot. The initial spot intensities for CyPet-FliN were divided by this number to estimate the number of fluorescent molecules per spot.

## SUPPLEMENTAL MATERIAL

Figure S1 CyPet-MotB stoichiometry and linear relationship between exposure time and fluorescence intensity. The Gaussian fit on the kernel density estimation peaks at 745 counts. The standard deviation is ± 112 counts. Inset shows linear relationship between intensities of CyPet-MotB motors and exposure times. The laser power was kept identical for each data point. Download Figure S1, PDF file, 0.3 MB

Figure S2 CyPet-FliN distribution in *cyPet-fliN*/Δ*cheY*/CheY^D57A^ strain (CCW motors). Gaussian kernel distribution estimation peaked at 143 ± 30 molecules. Download Figure S2, PDF file, 0.2 MB

Video S1 Tethered JPA810 cells. A fluorescent spot is visible at the center of rotation. Exposure time, 40 ms; field of view, 6 by 6 µm. Download Video S1, AVI file, 4.2 MB

Video S2 Moving CyPet-FliN spots in JPA811 cells. The cells are stuck to the coverslip. Exposure time, 40 ms; field of view, 6 by 6 µm. Download Video S2, AVI file, 3.7 MB

Video S3 Moving CyPet-FliN spots in JPA810 cells. The cells are stuck to the coverslip. Exposure time, 40 ms; field of view, 6 by 6 µm. Download Video S3, AVI file, 3.7 MB
