# Supplementary figures and images for: Stoichiometry and Turnover of the Bacterial Flagellar Switch Protein FliN
Source: mBio. 2014 Jul 1;5(4):e01216-14. doi: 10.1128/mBio.01216-14 (PMC4161238; doi:10.1128/mBio.01216-14)

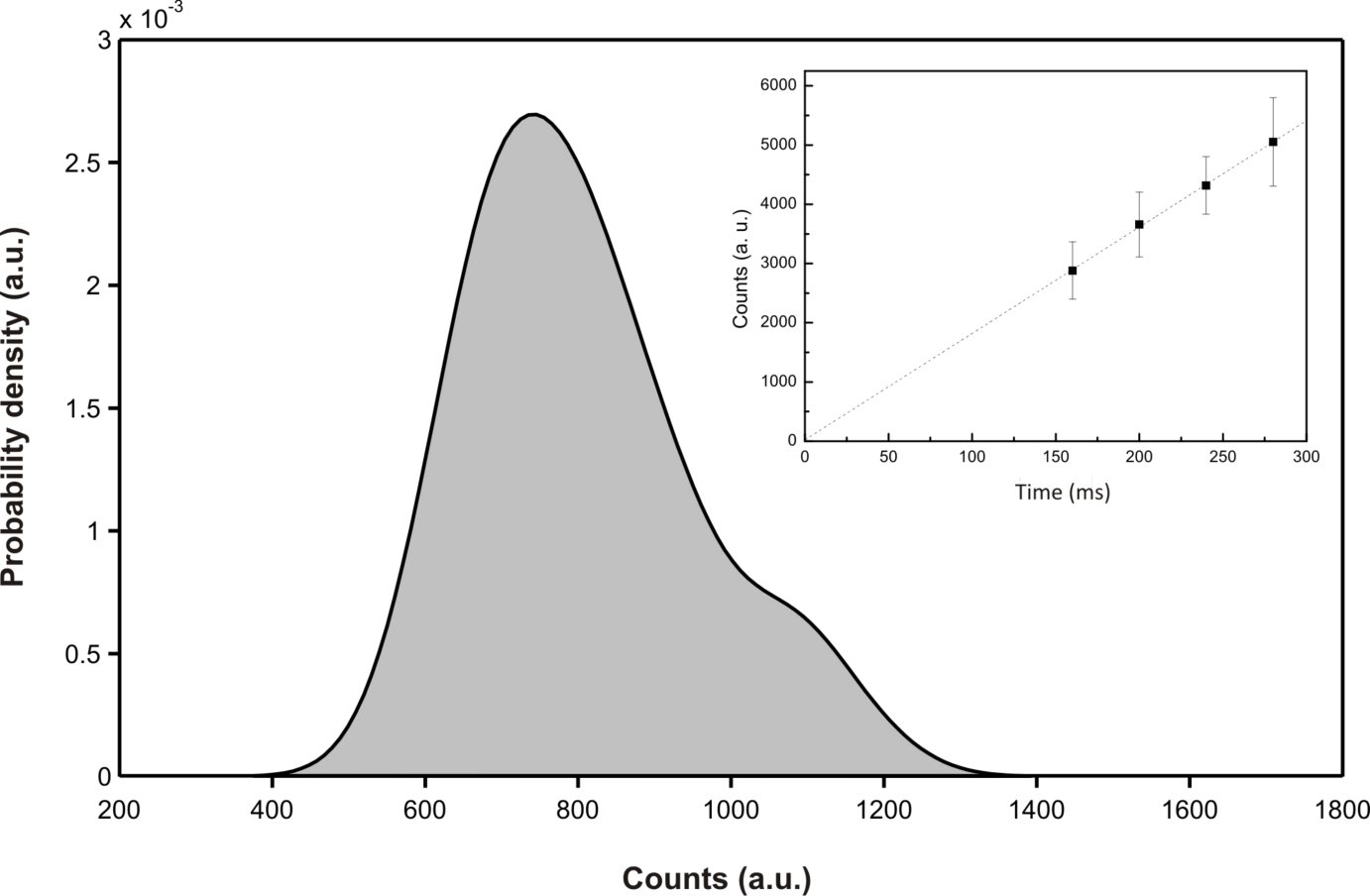

Supplement: Figure S1 — CyPet-MotB stoichiometry and linear relationship between exposure time and fluorescence intensity. The Gaussian fit on the kernel density estimation peaks at 745 counts. The standard deviation is ± 112 counts. Inset shows linear relationship between intensities of CyPet-MotB motors and exposure times. The laser power was kept identical for each data point. Download [file mbo003141894sf01.pdf]

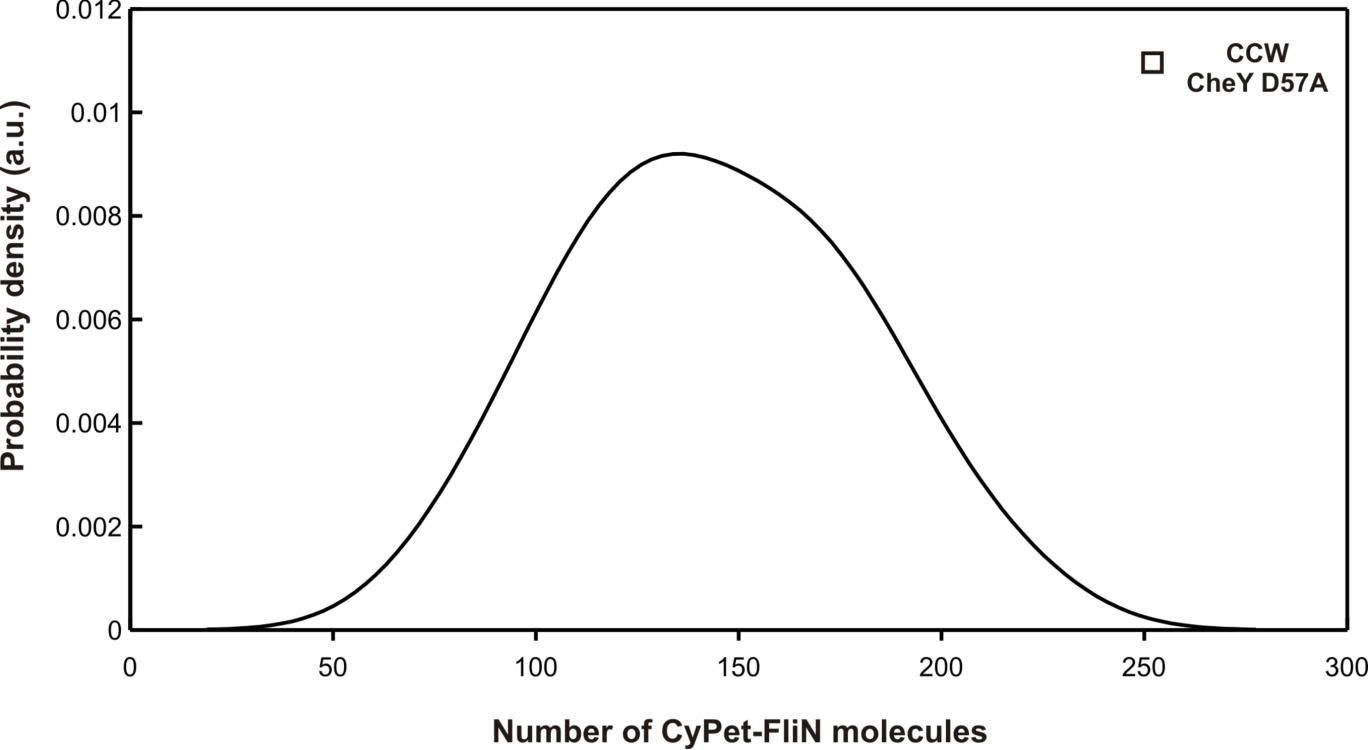

Supplement: Figure S2 — CyPet-FliN distribution in cyPet-fliN/ΔcheY/CheYD57A strain (CCW motors). Gaussian kernel distribution estimation peaked at 143 ± 30 molecules. Download [file mbo003141894sf02.pdf]
